# Measurement of Nutrition Environments in Grocery Stores, Convenience Stores, and Restaurants in the Lower Mississippi Delta

**DOI:** 10.5888/pcd17.190293

**Published:** 2020-03-12

**Authors:** Jessica L. Thomson, Melissa H. Goodman, Alicia S. Landry

**Affiliations:** 1 US Department of Agriculture, Agricultural Research Service, Stoneville, Mississippi; 2 Department of Family and Consumer Sciences, University of Central Arkansas, Conway, Arkansas

## Abstract

**Introduction:**

The Lower Mississippi Delta is characterized by several factors associated with poor diet quality. Our objective was to measure local nutrition environments of pregnant women and their infants who participated in a nutrition and physical activity intervention to assess environmental exposures potentially influencing their dietary habits.

**Methods:**

We measured the nutrition environments of 5 towns in which participants resided by using the Nutrition Environment Measures Survey for grocery stores, convenience stores, full-service restaurants, and fast food restaurants. We used general linear models to test for differences in ratio scores, calculated by dividing each food outlet score by the maximum score possible, among food outlet classes and subclasses.

**Results:**

Mean total ratio scores (expressed as percentages) for the Nutrition Environment Measures Survey among 4 classes of food outlets were significantly different from one another except for convenience stores and full-service restaurants. On average, grocery stores (n = 11) had 54% of maximum points possible, followed by full-service restaurants (21%; n = 50), convenience stores (16%; n = 86), and fast food restaurants (8%; n = 119). We found no significant differences in mean total ratio scores among convenience store subclasses. For fast food restaurant subclasses, stand-alone restaurants (n = 81) had 19% of maximum points possible, significantly higher than grocery store delicatessens (6%; n = 8), corner stores that sold fast food (3%; n = 5), and gas stations that sold fast food (4%; n = 25).

**Conclusion:**

These 5 Lower Mississippi Delta towns scored low on nutrition environment measures associated with healthful eating. Behavioral interventions designed to bring about positive changes in dietary habits of rural residents are needed; however, effects may be minimal if environmental factors supportive of healthful eating are not available.

**Summary**

**What is already known on this topic?**

The sociodemographic characteristics of the Lower Mississippi Delta population are linked to poor diet quality. Reproductive-age women residing in this region are at increased risk for adverse maternal and infant outcomes associated with suboptimal maternal nutrition.

**What is added by this report?**

The 5 Lower Mississippi Delta towns included in this study scored low on nutrition environment measures associated with healthful eating.

**What are the implications for public health practice?**

Assessment of nutrition environments before conducting dietary interventions in rural communities is necessary to first ascertain the presence of environmental supports that facilitate healthful eating.

## Introduction

The Mississippi River Delta region is among the most socioeconomically disadvantaged areas of the United States; the region has poorer health outcomes than non-Delta counties in the same states and the nation (1). Furthermore, counties in the Delta region have lower scores for factors contributing to a healthy food environment (eg, access to healthy foods, food security) than either non-Delta counties or the nation (1). Compounding these circumstances, reproductive-age women residing in this region are at increased risk for adverse outcomes associated with suboptimal maternal nutrition (2,3), including maternal and infant mortality, preterm birth, and low infant birth weight (4,5). For optimal health, consuming nutritious foods is essential to prevent and ameliorate nutrition-related health conditions, such as obesity, type 2 diabetes, and cardiovascular disease (6). Accessibility (the location of healthful food outlets near neighborhoods, particularly neighborhoods in low-income or rural areas), availability (healthful options in local food outlets), and affordability (reasonable prices) of nutrient-dense food resources are essential to facilitate adoption of a healthful diet (7–9).

From March 2013 through May 2016, we conducted the Delta Healthy Sprouts Project among pregnant women and their infants residing in the rural Lower Mississippi Delta. We designed the project to compare the effect of 2 maternal, infant, and early childhood home visiting curricula on health behaviors of women and their infants (10). Despite the project’s focus on nutrition intervention, early results indicated that the women’s baseline diet quality was poor and did not improve in either the gestational or postnatal periods (11,12). These results were concerning because the women’s unhealthy dietary habits may have adversely affected their infants’ dietary intake (13). Therefore, we conducted an exploratory ancillary investigation, the Delta Food Outlets Study, to measure local nutrition environments to which these women and their infants were exposed to assess environmental exposures potentially influencing their dietary habits. Such information is important to inform future nutrition interventions conducted in the same region of the country with similar populations.

## Methods

### Study setting

The Delta Food Outlets Study was an observational ancillary study to the Delta Healthy Sprouts Project; the ancillary study collected data on food outlets in 5 towns in which Delta Healthy Sprouts participants resided. The study was approved and classified as exempt by the institutional review board of Delta State University. Data collection occurred from March 2016 through September 2018.

### Food outlet identification and classification

We identified grocery stores by referencing 2 sources: the US Department of Agriculture (USDA) Food and Nutrition Service Supplemental Nutrition Assistance Program (SNAP) retailer locator (14), which lists SNAP retailers by state and location, and the Mississippi State Department of Health Restaurant and Food Facility Inspections website (15). We identified convenience stores by referencing 3 sources: the SNAP retailer locator (14); the B2B Yellow Pages website (16), which provides a search engine for business type and city/state location; and lists of current privilege licenses (licenses allowing a person to do business in the city for a profit) obtained from city clerks. Restaurants, which cannot operate without a current license and passing an annual inspection, were identified by referencing the Mississippi State Department of Health Restaurant and Food Facility Inspections website (15).

We classified food outlets by using operational definitions provided by the Economic Research Service in the documentation of its Food Environment Atlas (17). Briefly, grocery stores were defined as supermarkets and smaller grocery stores primarily engaged in retailing a general line of food, such as canned and frozen foods; fresh fruits and vegetables; and fresh and prepared meats, fish, and poultry. Convenience stores were defined as stores primarily engaged in retailing a limited line of goods that generally includes milk, bread, soda, and snacks. Full-service restaurants were defined as restaurants that provide food service to patrons who order and are served while seated and pay after eating. Fast food restaurants were defined as restaurants that provide food services (excluding snack and nonalcoholic beverage bars) where patrons generally order or select items and pay before eating. We further subclassified convenience stores as gas stations, national chain dollar stores (Dollar General, Dollar Tree, Family Dollar, and Fred’s), national chain pharmacies (CVS Pharmacy, Walgreens, and Getwell Drug and Dollar) or corner stores (all others). In addition to stand-alone chain restaurants and local fast food restaurants, grocery stores selling fast food (delicatessens), corner stores selling fast food, and gas stations selling fast food also were classified as fast food restaurants. Hence, grocery stores, corner stores, and gas stations selling fast food were measured twice: first to capture data on the nutritional aspects of the store environment and second to capture data on the nutritional aspects of the fast food environment. Before measurement, we visited all identified outlets to ensure that they were open and sold food.

### Measures

We measured the nutrition environments of food outlets by using the Nutrition Environment Measures Survey (NEMS) for grocery stores (NEMS–S), convenience stores (NEMS–CS), and restaurants (NEMS–R) (18). NEMS–S is a validated observational measure of retail store nutrition environments; it focuses on the availability of healthful food choices and quality fresh produce and compares the price of healthful options with the price of less healthful options in 11 common categories (19). NEMS–CS expanded NEMS-S to include additional measures for frozen and canned fruits and vegetables and quantities of fresh produce (20). Points are awarded according to the availability of healthful or more healthful options (eg, fresh fruits and vegetables, lower-fat ground beef), price comparisons between more healthful and less healthful options (eg, 100% fruit juice vs juice drink), and quality of fresh produce. Scores for the 3 scales — availability, price, and quality — are summed to create total scores for the NEMS–S and NEMS–CS. NEMS–R is a validated observational measure of restaurants designed to assess the relative healthfulness of foods and beverages available on main menus and children’s menus; it focuses on availability, facilitators, and supports for healthful eating; barriers to healthful eating; pricing; and signage/promotion (21). For food items, points are awarded according to availability of more healthful options (eg, lower-calorie/lower-fat entrées, fruit without added sugar, nonfried vegetables without added sauce) and price comparisons between more healthful and less healthful entrées. Scores for 4 scales – healthful options, facilitators for healthful eating, barriers to healthful eating, and children’s menu – are summed to create the total score for NEMS–R. For this study, we did not include points for the children’s menu in the total score so that all restaurants (those with and without children’s menus) were measured by using the same set of scales. However, we did compare scores for children’s menus at full-service and fast food restaurants. We scored food outlets by using algorithms provided for the tools; we modified the algorithms (Table 1) to reflect that water is a more healthful beverage choice than artificially sweetened soda and to decrease emphasis on some low-fat foods because they tend to have more sugar than their regular-fat counterparts (22).

**Table 1:** Differences Between the Original Scoring Algorithms of the Nutrition Environment Measures and the Revised Scoring Algorithms Used for the Delta Food Outlets Study, 2016–2018

Scale/Item	Points	
	Original	Revised
**Grocery Stores**		
**Availability**		
Frozen dinners	1 or 2 reduced-fat types available = 1 point	2 reduced-fat types available = 2 points
		1 reduced-fat type available = 1 point
Beverages	Diet soda available = 1 point	Bottled water available = 1 point
Cereal	No points for availability of >2 lower-sugar varieties	>2 Lower-sugar varieties available = 1 point
Total	Possible range, 0 to 30 points	Possible range, 0 to 31 points
**Price**		
Frozen dinners	No points for equal prices of low-fat and regular-fat dinners	Equal prices for low-fat and regular-fat dinners = 1 point
Baked goods	No points for equal prices of low-fat and regular-fat goods	Equal prices for low-fat and regular-fat goods = 1 point
Beverages	Lower price for diet soda than for regular soda = 2 points	Lower price for water than for regular soda = 2 points
		Equal prices for water and regular soda = 1 point
		Higher price for water than for regular soda = −1 point
		Lower price for 100% juice than for juice drink = 2 points
		Equal prices for 100% juice and juice drink = 1 point
Bread	No points for equal prices of whole-wheat bread and refined-grain bread	Equal prices for whole-wheat bread and refined-grain bread = 1 point
Baked chips	No points for equal prices of baked chips and regular chips	Equal prices for baked chips and regular chips = 1 point
Cereal	No points for equal prices of lower-sugar cereal and higher-sugar cereal	Equal prices for lower-sugar cereal and higher-sugar cereal = 1 point
Total	Possible range, −9 to 18 points	Possible range, −10 to 20 points
**Quality**	Possible range, 0 to 6 points	Possible range, 0 to 6 points (no change from original)
**Total**	Possible range, −9 to 54 points	Possible range, −10 to 57 points

**Convenience Stores**		
**Availability**		
Ground beef	2 or 3 Varieties <10% fat = 1 point	≥1 variety <10% fat = 2 points
	>3 Varieties <10% fat = 2 points	
Beverages	Diet soda available = 1 point	Bottled water available = 1 point
	Non-carbonated, no-calorie or low-calorie available = 1 point	
100-Calorie snacks	Available = 2 points	Available = 0 points
Cereal	No points for >2 lower-sugar varieties	>2 Lower-sugar varieties = 1 point
Total	Possible range, 0 to 34 points	Possible range, 0 to 31 points
**Price**		
Ground beef	No points for equal prices of lower-fat ground beef and higher-fat ground beef	Equal prices for lower-fat ground beef and higher-fat ground beef = 1 point
Hot dogs	No points for equal prices of lower-fat hot dogs and higher-fat hot dogs	Equal prices for lower-fat hot dogs and higher-fat hot dogs = 1 point
Frozen dinners	No points for equal prices of lower-fat dinners and higher-fat dinners	Equal prices for lower-fat dinners and regular-fat dinners = 1 point
Baked goods	No points for equal prices of lower-fat goods and higher-fat goods	Equal prices for lower-fat goods and regular-fat goods = 1 point
Beverages	Lower price for diet soda than for regular soda = 2 points	Lower price for water than for 100% juice = 2 points
	No points for equal prices of water and 100% juice	Equal prices for water and 100% juice = 1 point
		Higher price for water than for 100% juice = −1 point
Bread	No points for equal prices of whole-wheat bread and refined-grain bread	Equal prices for whole-wheat bread and refined-grain bread = 1 point
Baked chips	No points for equal prices of baked chips and regular chips	Equal prices for baked chips and regular chips = 1 point
Cereal	No points for equal prices of lower-sugar cereal and higher-sugar cereal	Equal prices for lower-sugar cereal and higher-sugar cereal = 1 point
Total	Possible range, −9 to 18 points	Possible range, −9 to 20 points
**Quality**	Possible range, 0 to 6 points	Possible range, 0 to 6 points (no change from original)
**Total**	Possible range, −9 to 58 points	Possible range, −9 to 57 points

**Restaurants**		
**Availability of healthful options**		
Low-fat or fat-free dressing	1 to 3 Choices = 1 to 3 points	No points
Fruit^a^	≥1 choice = 1 point	1 choice = 1 point, 2 to 4 choices = 2 points, ≥5 choices = 3 points
Vegetable^b^	≥1 choice = 1 point	1 choice = 1 point, 2 to 4 choices = 2 points, ≥5 choices = 3 points
Bottled water	No points for availability	Available = 1 point
Total	Possible range, 0 to 15 points	Possible range, 0 to 17 points
**Facilitators for healthful eating**		
Menu nutrition information or healthy entrée identified	Presence of either = 1 point	Presence = 1 point per facilitator
Signage highlighting healthful options or healthy eating encouraged	Presence of either = 1 point	Presence = 1 point per facilitator
Total	Possible range, 0 to 8 points	Possible range, 0 to 10 points
**Barriers to healthful eating**		
Shared entrée	No points for extra cost	Extra cost = −1 point
Signage, unhealthful eating encouraged or overeating encouraged	Presence of either = −1 point	Presence = −1 point per barrier
Total	Possible range, −5 to 0 points	Possible range, −7 to 0 points
**Children’s menu**	Possible range, −3 to 9 points	Possible range, −3 to 9 points (no change from original)
**Total^c^**	Possible range, −5 to 21 points	Possible range = −7 to 27 points

a Without added sugar.

b Nonfried and without added sauce.

c Sum of points for availability, facilitators, and barriers scales; does not include points from children’s menu scale.

Before data collection, senior researchers and research assistants (data collectors) completed a NEMS online training course, which included fieldwork, and were certified as NEMS raters (18). We recreated the NEMS tools as electronic surveys by using Snap Surveys software version 11.20 (Snap Surveys Ltd). All data were collected via tablets loaded with Snap Surveys software and stored on the Snap WebHost, an online mobile and secure survey management system. For quality assurance purposes, we randomly selected 25% of the food outlets for duplicate measurement. Discrepancies between duplicate measurements were discussed and resolved.

### Data analyses

We performed statistical analyses by using SAS version 9.4 (SAS Institute Inc). To allow for comparisons among food outlet classes, we transformed NEMS scores into ratio scores by dividing each food outlet score by the maximum score possible. The use of ratio scores was necessary because each NEMS tool has a different possible score range (NEMS–S, −10 to 57; NEMS–CS, −9 to 57; NEMS–R, −7 to 27). The higher the ratio score, the more healthful the nutrition environment. We used general linear models with normal distributions and custom contrasts to test for differences in ratio scores among the food outlets. Class was modeled as a main effect, and subclass was nested within class. The NEMS–R scale was modeled with a β distribution because ratio scores represented true proportions (ie, no negative ratios nor ratios equal to the boundaries of 0 and 1). We visually inspected residual plots to confirm that residuals were approximately normally distributed. We computed least squares means and 95% confidence intervals from the general linear models, because they are adjusted for other factors in the model and because of the unequal sample sizes in the food outlet classes and subclasses. To correct for multiple testing, we adjusted the significance level of .05 by using a Bonferroni correction factor (ie, .05 of 6 comparisons = .008).

## Results

Of the 280 food outlets identified, 12 were closed (3 convenience stores and 9 restaurants), 1 refused to be measured (convenience store), and 1 was deemed unsafe (convenience store). The resulting analytic sample of 266 food outlets comprised 11 grocery stores, 86 convenience stores, 50 full-service restaurants, and 119 fast food restaurants (Table 2). The population of the 5 towns ranged from 1,750 to 32,612. The percentage of population that was African American ranged from 49% to 91%, and the percentage of population that lived below the federal poverty level ranged from 29% to 51%. At baseline, 11% of Delta Healthy Sprouts participants had passing diet quality scores (60%–72% of total points). The percentage of participants with passing scores who resided in towns with 4 or 5 grocery stores (11%) was approximately equal to that of participants who resided in towns with 0 or 1 grocery stores (12%).

**Table 2:** Characteristics of Towns Included in the Delta Food Outlet Study, 2016–2018

Characteristic	Town 1	Town 2	Town 3	Town 4	Town 5	Total
**Population size^a^**	32,612	12,346	4,254	2,484	1,750	—
Percentage African American^a^	80	49	69	86	91	—
Percentage living below federal poverty level^a^	36	29	32	41	51	—
**No. of food outlets**						
Grocery store	5	4	1	1	0	11
Convenience store	48	18	10	6	4	86
Full-service restaurant	28	16	5	1	0	50
Fast food restaurant	60	40	8	7	4	119

a 2012–2016 American Community Survey 5-year estimates (23).

Of 370 items analyzed in grocery stores, discrepancies in duplicate measurements ranged from 1 to 10 items (0.3%–2.7%) per store; the most common discrepancy was availability of diet soda. Of 389 items examined in convenience stores, discrepancies in duplicate measurements ranged from 0 to 7 items (0%–1.8%) per store; the most common discrepancy was food brand. Of 142 items examined in restaurants, discrepancies in duplicate measurements ranged from 0 to 8 items (0%–5.6%) per restaurant; the most common discrepancy was hours of operation.

Mean total ratio scores for the 4 classes of food outlets were significantly different from one another except for convenience stores and full-service restaurants (Table 3). Grocery stores had the highest mean total ratio score (0.54) followed by full-service restaurants (0.21), convenience stores (0.16), and fast food restaurants (0.08). We found no significant differences for mean total ratio scores among convenience store subclasses. For fast food restaurant subclasses, stand-alone restaurants had a significantly higher mean total ratio score (0.19) than grocery store delicatessens (0.06), corner stores that sold fast food (0.03), and gas stations that sold fast food (0.04).

**Table 3:** Nutrition Environment Measures Survey Scores for Food Outlets (N = 266) by Class and Subclass of Food Outlet, Delta Food Outlets Study, 2016–2018

Class or Subclass of Food Outlet and Type of Scale	Possible Score Range	No. of Outlets	Original Score		Ratio Score, Least Squares Mean (95% CI)^a^
			Mean (SD)	Minimum–Maximum	
**Total**					
**Class**					
Grocery store	−10 to 57	11	30.5 (7.5)	22 to 41	0.54 (0.47 to 0.60)^b^
Convenience store	−9 to 57	86	8.1 (3.2)	2 to 14	0.16 (0.13 to 0.19)^c^
Full-service restaurant	−7 to 27	50	5.6 (3.5)	−2 to 18	0.21 (0.18 to 0.24)^c^
Fast food restaurant	−7 to 27	119	3.9 (4.0)	−2 to 19	0.08 (0.05 to 0.12)^d^
**Convenience store subclass**					
Corner store	−9 to 57	21	6.6 (2.9)	2 to 14	0.12 (0.07 to 0.16)^b^
Gas station	−9 to 57	40	7.2 (2.8)	2 to 14	0.13 (0.09 to 0.16)^b^
National chain dollar store	−9 to 57	21	10.7 (2.3)	4 to 14	0.19 (0.14 to 0.24)^b^
National chain pharmacy	−9 to 57	4	12.0 (2.5)	9 to 14	0.21 (0.10 to 0.32)^b^
**Fast food subclass **					
Restaurant stand-alone	−7 to 27	81	5.2 (4.1)	−1 to 19	0.19 (0.17 to 0.22)^b^
Grocery store delicatessen^e^	−7 to 27	8	1.8 (1.9)	−1 to 5	0.06 (−0.01 to 0.14)^c^
Corner store^f^	−7 to 27	5	0.8 (1.9)	−1 to 4	0.03 (−0.07 to 0.13)^c^
Gas station^f^	−7 to 27	25	1.0 (1.8)	−2 to 5	0.04 (−0.01 to 0.08)^c^

**Type of Scale**					
**Availability scale**					
Grocery store	0 to 31	11	19.6 (5.7)	12 to 28	0.63 (0.58 to 0.68)^b^
Convenience store	0 to 31	86	5.6 (3.0)	1 to 11	0.21 (0.19 to 0.23)^c^
Convenience store subclass					
Corner store	0 to 31	21	3.9 (1.5)	1 to 8	0.12 (0.10 to 0.15)^b^
Gas station	0 to 31	40	4.1 (1.5)	1 to 7	0.13 (0.11 to 0.15)^b^
National chain dollar store	0 to 31	21	9.6 (1.8)	4 to 11	0.31 (0.28 to 0.35)^c^
National chain pharmacy	0 to 31	4	10.0 (1.4)	8 to 11	0.32 (0.25 to 0.41)^c^
**Price scale**					
Grocery store	−10 to 20	11	5.0 (3.5)	−1 to 9	0.25 (0.20 to 0.30)^b^
Convenience store	−9 to 20	86	2.3 (1.6)	−2 to 5	0.10 (0.08 to 0.13)^c^
Convenience store subclass					
Corner store	−9 to 20	21	2.3 (1.9)	0 to 4	0.11 (0.08 to 0.15)^b^^,^ c
Gas station	−9 to 20	40	2.9 (1.3)	0 to 5	0.14 (0.12 to 0.17)^b^
National chain dollar store	−9 to 20	21	1.1 (1.5)	−1 to 4	0.05 (0.02 to 0.09)^c^
National chain pharmacy	−9 to 20	4	2.0 (2.7)	−2 to 4	0.10 (0.01 to 0.19)^b^^,^ c
**Healthful options scale**					
Full-service restaurant	0 to 17	50	5.5 (2.7)	1 to 14	0.32 (0.28 to 0.37)^b^
Fast food restaurant	0 to 17	119	3.7 (3.1)	0 to 12	0.13 (0.08 to 0.18)^c^
Fast food subclass					
Restaurant stand-alone	0 to 17	81	4.8 (3.1)	0 to 12	0.28 (0.25 to 0.32)^b^
Grocery store delicatessen^e^	0 to 17	8	1.8 (1.2)	0 to 3	0.10 (−0.01 to 0.21)^c^
Corner store^f^	0 to 17	5	0.8 (1.3)	0 to 3	0.05 (−0.03 to 0.19)^c^
Gas station^f^	0 to 17	25	1.4 (1.4)	0 to 5	0.08 (0.02 to 0.14)^c^
**Facilitators for healthful eating scale** g					
Full-service restaurant	0 to 10	50	0.7 (1.1)	0 to 6	—
Fast food restaurant	0 to 10	119	0.9 (1.3)	0 to 7	—
Fast food subclass					
Restaurant stand-alone	0 to 10	81	1.2 (1.5)	0 to 7	—
Grocery store delicatessen^e^	0 to 10	8	0.4 (0.7)	0 to 2	—
Corner store^f^	0 to 10	5	0.2 (0.5)	0 to 1	—
Gas station^f^	0 to 10	25	0.5 (0.1)	0 to 2	—
**Barriers to healthful eating scale** g					
Full-service restaurant	−7 to 0	50	−0.5 (0.8)	−3 to 0	—
Fast food restaurant	−7 to 0	119	−0.7 (0.9)	−3 to 0	—
Fast food subclass					
Restaurant stand-alone	−7 to 0	81	−0.7 (1.0)	−3 to 0	—
Grocery store delicatessen^e^	−7 to 0	8	−0.4 (0.5)	−1 to 0	—
Corner store^f^	−7 to 0	5	−0.2 (0.5)	−1 to 0	—
Gas station^f^	−7 to 0	25	−0.8 (0.8)	−2 to 0	—
**Children’s menu scale** h					
Full-service restaurant	−3 to 9	26	0.0 (1.4)	−1 to 4	0.00 (−0.07 to 0.07)^b^
Fast food restaurant	−3 to 9	41	1.8 (1.7)	−2 to 5	0.20 (0.14 to 0.25)^c^

Abbreviations: CI, confidence interval; SD, standard deviation.

a Nutrition Environment Measures Survey scores were translated into ratio scores by dividing the score for each food outlet by the maximum score possible. Least squares means and associated 95% confidence intervals were computed from the general linear models used to test for significant differences among classes and subclasses of food outlets.

b,c,d Food outlet classes or subclasses with different letters are significantly different from one another at *P* < .003 based on general linear model results. Food outlet classes or subclasses with the same letters are not significantly different from one another.

e Dual classification as grocery store.

f Dual classification as convenience store.

g Differences in ratio score among classes and subclasses were not significant.

h None of the fast food restaurant subclasses had children’s menus except stand-alone restaurants.

For availability and price scales, mean ratio scores for grocery stores were significantly higher than scores for convenience stores (0.63 vs 0.21 and 0.25 vs 0.10, respectively). Among convenience store subclasses, mean ratio scores for the availability scale were significantly higher for national chain dollar stores and pharmacies (0.31 and 0.32, respectively) than for corner stores and gas stations (0.12 and 0.13, respectively). Mean ratio scores for the price scale were significantly higher for gas stations (0.14) than for national chain dollar stores (0.05). Because so few convenience stores sold fresh fruits or fresh vegetables, we did not compare convenience stores and grocery stores for the quality scale. Descriptively, all but 1 of the 11 grocery stores received the maximum score of 6 for the quality scale, whereas the one received a score of 5. Of the 86 convenience stores, 8 (4 corner stores and 4 gas stations) sold fresh produce; quality scores were 0 (n = 3), 4 (n = 4), and 6 (n = 1). Mean (standard deviation) quality scale scores were 5.9 (0.3) for grocery stores and 2.3 (2.1) for convenience stores.

For restaurants, the mean ratio score for the healthful options scale was significantly higher for full-service restaurants (0.32) than for fast food restaurants (0.13). Conversely, the mean ratio score for the children’s menu scale was significantly higher for fast food restaurants (0.20) than for full-service restaurants (0). Among fast food restaurant subclasses, the mean ratio score for the healthful options scale was significantly higher for stand-alone fast food restaurants (0.28) than for grocery stores with a delicatessen (0.10), corner stores (0.05), and gas stations (0.08). For the facilitators and barriers scales, we did not find significant differences in mean ratio scores between full-service restaurants and fast food restaurants and among fast food restaurant subclasses.

## Discussion

We presented nutrition environment measures of grocery stores, convenience stores, full-service restaurants, and fast-food restaurants located in towns in which Delta Healthy Sprouts participants resided. To our knowledge, this is the first study to directly compare nutrition environment measures among these 4 classes of food outlets in the rural Lower Mississippi Delta. Results indicate that nutrition environments may not have been conducive to effecting positive dietary changes in this cohort of rural, Southern, primarily African American women. Of the 4 food outlet classes, grocery stores had the most healthful nutrition environments. However, they only had 54% of the maximum score on average, which qualitatively equates to a failing grade on a 10-point grading scale. Fast food restaurants had the least healthful nutrition environments, having less than 10% of the maximum score on average, indicating that few if any healthful options were available at these outlets. Grocery stores also outperformed convenience stores on the availability and price scales, although again, both classes of outlets scored below 65% and 30%, respectively, of the maximum scores on average. These results indicate that availability of healthful foods was low, and the price of more healthful foods did not compare favorably with the price of less healthful foods. Full-service restaurants outperformed fast food restaurants on the healthful options scale, but the reverse was true for the children’s menu scale, for which fast food restaurants outperformed full-service restaurants. Again, scores were not optimal, achieving failing grades of less than 35% of maximum scores. Thus, although some classes of food outlets did outperform others, the nutrition environments of all classes were poor and need improvement to facilitate healthful food choices for residents in these communities.

In a study conducted in Alabama’s Black Belt region, mean NEMS–S total scores were 30 for grocery stores and 7 for convenience stores (possible range, −9 to 54) (24), similar to the scores in our study (31 and 8, respectively). Mean NEMS–S total scores were higher for grocery stores and convenience stores (36 and 13, respectively; possible range, −9 to 54) in a rural Minnesota community than in our study (25). Conversely, the mean NEMS–S total score (24; possible range, −9 to 54) was lower for grocery stores located in rural Montana counties, where half the stores measured were on an Indian reservation (26), than for grocery stores in our study. Mean NEMS–S total scores for grocery stores located in rural Washington towns varied from 15 to 35 (possible range, −8 to 62), whereas mean total scores for convenience stores varied from 3 to 4 (possible range, −8 to 62) (26). Collectively, results from these studies suggest that the retail nutrition environment in the rural United States is uniformly low; grocery and convenience stores score no more than two-thirds to less than one-quarter of maximum points for indicators of healthful nutrition environments.

Ko and colleagues (27) reported NEMS–R total scores for restaurants located in rural Washington towns; means varied from 4 to 23 for full-service restaurants and from 8 to 19 for fast food restaurants (possible range, −8 to 72). Proportionally, mean scores for rural Washington restaurants (0.05–0.32 for full-service, 0.11–0.26 for fast food) were somewhat similar to scores in our study (0.21 for full-service subclass, 0.03–0.19 for fast food subclass). Although nutrition environments of restaurants were measured in the rural Minnesota community (25), the authors did not report total or scale scores, so we cannot make direct comparisons with our study results. Pereira and colleagues noted that findings were mixed on whether full-service or fast food restaurants had healthier practices (25), similar to our findings that full-service restaurants scored significantly higher than fast food restaurants on the healthful options scale but significantly lower on the children’s menu scale. Results from these studies suggest that nutrition environments of restaurants located in rural communities are suboptimal.

Taken together, these results suggest that behavioral interventions targeting dietary habits of residents of rural communities will not be successful or have long-lasting effects without concurrent changes in local food environments. Retail food environments at both the community level (eg, presence of supermarkets) and the consumer level (eg, healthful affordable foods in convenience stores) are promising venues for effecting positive change in nutrition environments (28). However, results from our study suggest that the presence of grocery stores with suboptimal nutrition environments are not sufficient to improve the diet quality of community residents, because the percentage of participants with passing scores who resided in towns with 4 or 5 grocery stores (11%) was approximately equal to that of participants who resided in towns with 0 or 1 grocery stores (12%).

Additionally, all the towns in which participants resided contained at least 1 convenience store or fast food establishment, food outlets with the poorest nutrition environments. Thus, improving the nutrition environment of convenience stores and fast food establishments as well as teaching rural residents how to navigate in suboptimal food environments to make healthful food choices may prove more efficacious than improving access to grocery stores.

Nutrition environments of restaurants also should be targeted, given the increasing percentages of the population eating away from home, particularly at fast food restaurants (29). A rural communitywide program designed to improve nutrition environments of restaurants was successful at increasing healthful practices of restaurants enrolled in the program as well as restaurants that chose not to enroll (30). Concentrated efforts to improve access and availability within the rural food landscape is becoming increasingly important. It is essential that community partners leverage both public and private funding, such as that provided by the Healthy Food Financing Initiative and the Mid South Healthy Food Initiative, both of which work to increase or expand access to healthy foods in underserved communities. In the absence of environmental changes, it may be necessary to teach residents of underserved communities how to navigate their suboptimal nutrition environments to make more healthful food choices, thereby improving the quality of their diet.

Strengths of our study include the use of validated and objective tools to measure the nutrition environments of rural towns in the Lower Mississippi Delta, the relatively large number of food outlets measured, and the population studied — African American children and adults living in the rural South — who are at increased risk for poor dietary habits (31). Limitations include the nonrandom selection and small number of towns measured; these limitations may restrict the generalizability of the study’s results. Some components of the food environment (eg, farmers markets, roadside stands, vending machines) were not included. Additionally, our study results may not accurately reflect the nutrition environments of Delta Healthy Sprouts participants because of temporal differences (ie, the Delta Food Outlets was conducted after Delta Healthy Sprouts was completed). Despite these limitations, this study provides unique and valuable contributions on the potential effects of nutrition environment exposure on health. Additional analytic efforts will include the determination of food outlet clustering in these towns, particularly in relation to proximity to participants’ residences.

The food outlets located in the Lower Mississippi Delta towns included in this study scored low on nutrition environment measures associated with healthful eating. Although behavioral interventions designed to bring about positive changes in the dietary habits of rural residents are needed, effects may be minimal or not sustainable if environmental supports, such as food outlets with an abundance of healthful food choices, are not available.
